# EMPLOYMENT AS A FORM OF SOCIAL PARTICIPATION AMONG OLDER ADULTS: LINKS TO COGNITIVE AND FUNCTIONAL HEALTH

**DOI:** 10.1093/geroni/igad104.1558

**Published:** 2023-12-21

**Authors:** Peilin Yang, Linda Waite, Ashwin Kotwal

**Affiliations:** University of Michigan Ann Arbor, Ann Arbor, Michigan, United States; University of Chicago, Chicago, Illinois, United States; University of California San Francisco, San Francisco, California, United States

## Abstract

Within the active aging literature, studies on social participation and health concur that people who are better socially integrated and engage in social activities tend to have better physical, mental, and cognitive health. This study revisits the literature by aiming to address three primary knowledge gaps in prior literature: 1) we explicitly examine the change within the 5-year-interval in cognition, activities of daily living (ADL), instrumental activities of daily living (IADL) associated with social participation five years prior; 2) we examine diversity in participation’s association with not only cognitive function, but also ADL and IADL, which we lack knowledge of; 3) we conceptualize employment in later life as a kind of social participation, a part of older adults’ lives that is overlooked in the social participation literature. We also examine whether the relationship between social participation and cognition, ADL, and IADL is the same for men and women, and for those employed and those not employed. The study finds that neighborhood participation at a high level indicates worse cognitive, ADL, and IADL outcomes 5 years later, and a higher level of neighborhood participation is more indicative of worse cognitive outcomes for men than for women. Full-time employment predicts better cognitive, ADL, and IADL outcomes 5 years later. We also find evidence that full-time work creates a stronger buffer against cognitive decline, developing ADL difficulties, and IADL difficulties, even among older adults who socialize with family and friends, participate in the community, and the neighborhood at a high level, respectively.

